# SAS-CARE-1 study: stroke topography and polysomnography-based respiratory sleep characteristics

**DOI:** 10.1007/s11325-025-03540-8

**Published:** 2025-12-05

**Authors:** Irina Filchenko, Anne-Kathrin Brill, Martijn P. J. Dekkers, Sébastien Baillieul, Andrea Seiler, Mauro Manconi, Roland Wiest, Corrado Bernasconi, Markus H. Schmidt, Claudio L. A. Bassetti

**Affiliations:** 1 Department of Neurology, Bern University Hospital (Inselspital), University of Bern, Freiburgstrasse 16, Bern, 3010 Switzerland; 2 Graduate School of Health Sciences, University of Bern, Mittelstrasse 43, Bern, 3012 Switzerland; 3 Department of Pulmonary Medicine and Allergology, Inselspital, Bern University Hospital, University of Bern, Freiburgstrasse 16, Bern, 3010 Switzerland; 4 Grenoble Alpes University, HP2 Laboratory, INSERM U1300, Grenoble Alpes University Hospital, Grenoble, France; 5 Sleep Medicine Unit, Neurocenter of the Southern Switzerland, Regional Hospital of Lugano, Lugano, Switzerland; 6 Faculty of Biomedical Sciences, Università della Svizzera Italiana, Lugano, Switzerland; 7 Department of Neuroradiology, University Hospital, Inselspita, Bern, Switzerland; 8 Medical Faculty, University of Bern, Bern, Switzerland; 9 Schlaf-Wach-Epilepsie-Zentrum SWEZ, Universitätsklinik für Neurologie, Inselspital, Freiburgstrasse 10, Bern, 3010 Switzerland

**Keywords:** Sleep-disordered breathing, Ischemic stroke, Obstructive apnea index, Central sleep apnea index, Polysomnography

## Abstract

**Purpose:**

Sleep-disordered breathing (SDB) is a risk factor of ischemic stroke but can also appear “de novo” after the acute brain damage. The link between SDB and stroke topography, which remains controversial, was assessed in this study.

**Methods:**

This is a post-hoc analysis of the multicenter prospective SAS-CARE-1 study, which included 259 patients with acute stroke or transient ischemic attack. Assessments comprised demographics, medical history, and brain magnetic resonance imaging (MRI) at admission. In 101 patients with acute stroke, MRI-based stroke topography was binary coded for the involvement as supra- (cortical and/or subcortical) and/or infratentorial (brainstem and/or cerebellar) locations. Polysomnography was performed at admission (*n* = 101) and at 3 months post-stroke (*n* = 72).

**Results:**

A linear regression with adjustments for age and sex identified an association of brainstem stroke with a higher obstructive apnea index (OAI; 9.91 events/h for brainstem stroke versus no brainstem involvement, *p* = 0.003) at acute stroke, but not at 3 months post-stroke. Correspondingly, the decrease of OAI from acute to 3 months post-stroke was associated with brainstem (-10.72 events/h, *p* = 0.011) stroke with adjustment for age and sex. However, this association was not significant with an additional adjustment for baseline OAI. There were no other significant associations.

**Conclusion:**

This study confirms the previously reported association between brainstem stroke and obstructive apneas. Further research is needed to elucidate the link between focal brain damage and respiratory control during sleep.

**Graphical abstract:**

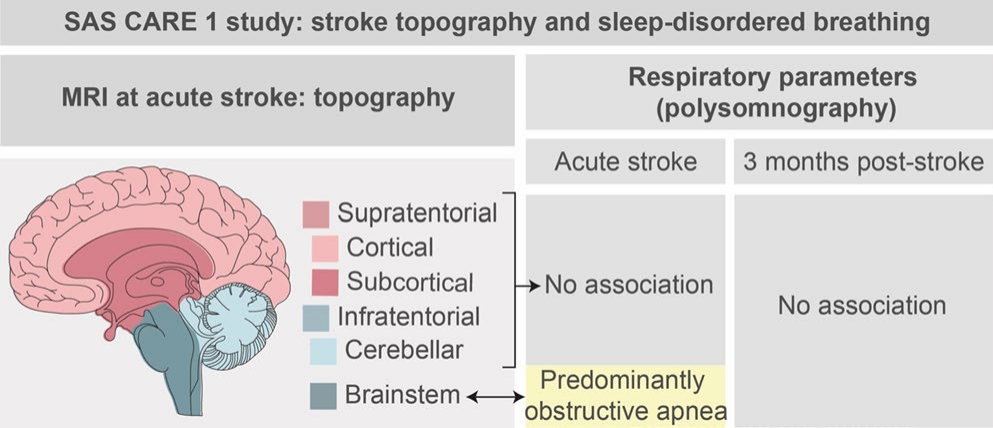

**Supplementary Information:**

The online version contains supplementary material available at 10.1007/s11325-025-03540-8.

## Introduction

Sleep-disordered breathing (SDB) is a common sleep-wake disorder with a global prevalence of 50% as defined by the apnea-hypopnea index (AHI) ≥5/h [1]. It also affects two thirds of stroke patients in both the acute and chronic phases [2]. The pathophysiological sequelae of SDB, including intermittent hypoxia, inflammation, and sympathetic hyperactivation, reinforce its role as a vascular risk factor [3].

Stroke itself can be a risk factor for new-onset SDB or for exacerbating pre-existing SDB. Indeed, specific brain lesions may disrupt ventilatory control or impair upper-airway muscle tone, leading to either central sleep apnea (CSA) or obstructive sleep apnea (OSA) [3]. Furthermore, comorbidities, body position and sleep architecture changes might affect the polysomnographic presentation of stroke-associated SDB. Few studies matched SDB phenotype and stroke topography in its acute phase using both respiratory polygraphy (RPG) [4–10] or conventional polysomnography (PSG) [11–14]. The advantage of PSG compared to RPG lies in a more complete detection of respiratory events due to the additional evaluation of sleep-wake (e.g., microarousals) [15]. While few RPG-based studies showed the association of predominantly central SDB with stroke lesions in prefrontal/frontal, thalamic/hypothalamic regions, deep MCA territory [4], or infratentorial locations [5, 9], other RPG-based studies did not confirm these findings [6–8, 10, 16]. Although no significant associations between stroke topography and SDB phenotypes were identified in three PSG-based studies [12–14], brainstem lesions were rather associated with an obstructive than a central phenotype.

Considering the current uncertainty in the literature, the current study was planned with the hypothesis that lesions in specific brain structures may correlate with type and severity of SDB. The primary aim of this analysis was to explore the association between stroke topography and SDB phenotype in the acute stroke. The secondary aim was to investigate whether such an association persists over time.

## Methods

### Study population

The current analysis included a subset of the prospective multicenter SAS CARE 1 study [17] and is reported according to STROBE guidelines. The study was conducted in Switzerland (Bern, Lugano), Germany (Münster) and Italy (Milan). The current analysis included only data from Swiss centers due to technical availability. The protocol (ClinicalTrials.gov identifier: NCT01097967) was approved by the local ethics committees. The study was conducted in compliance with the Declaration of Helsinki and Good Clinical Practice. All study participants provided written informed consent prior to participation.

### Study assessments

Details regarding the design of the SAS-CARE 1 study have been described previously [17]. The inclusion criteria were the following: 1) acute ischemic stroke or transient ischemic attack diagnosed within 2 days from symptom onset; 2) age 18-75 years. The exclusion criteria were the following: 1) unstable clinical condition; 2) SDB treatment currently or during the last 3 months before stroke; 3) nonischemic events (intracerebral/subarachnoid hemorrhage); and 4) any condition that may interfere with the acceptance of SDB treatment.

**Fig. 1:**
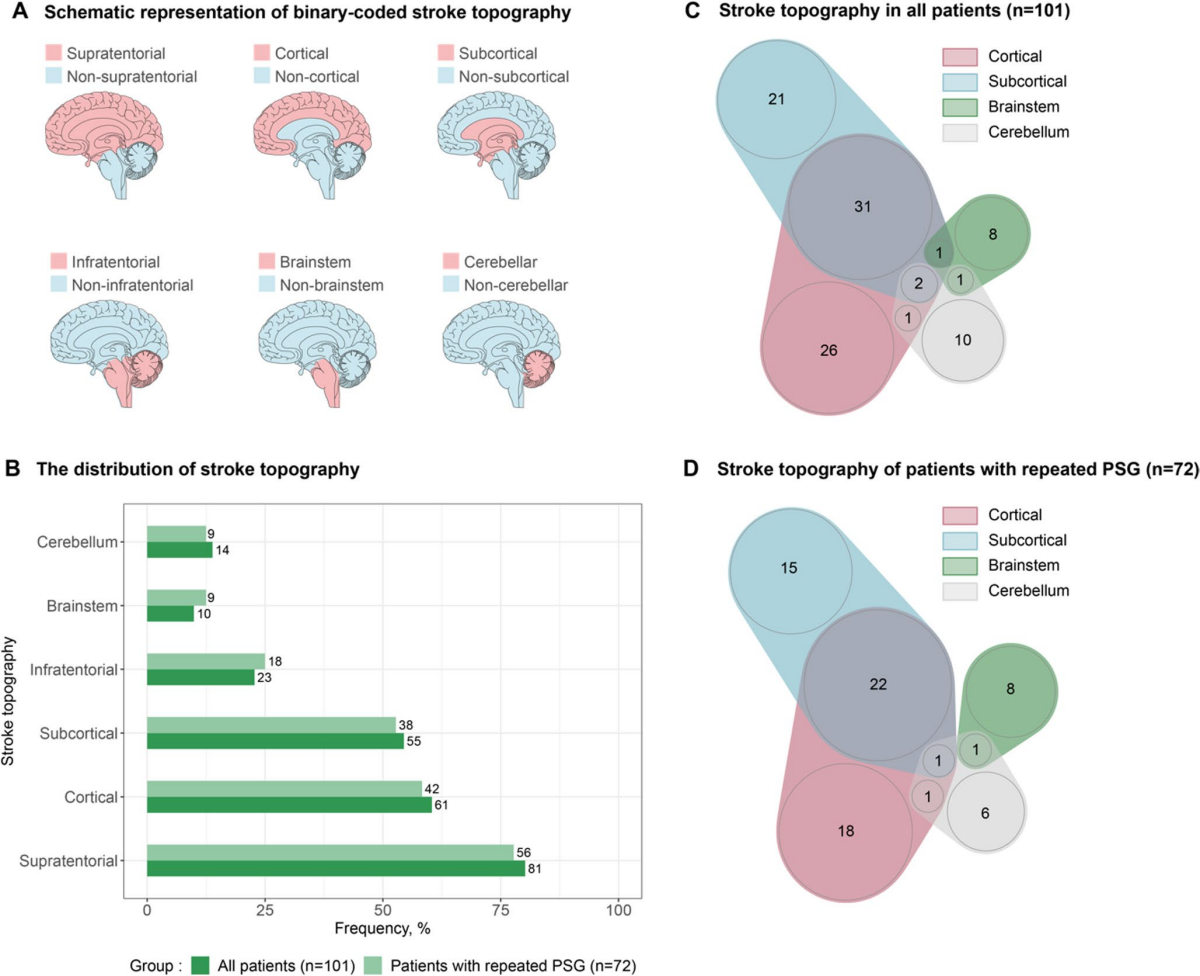
Stroke topography. **A**. Schematic representation of binary-coded stroke topography **B**. The distribution of stroke topography with the consideration that stroke could affect multiple locations in one study participant. There were no differences in the distribution of stroke topography between all patients (*n* = 101) and the patients with repeated polysomnography (*n* = 72) according to fisher exact test. **C**. Venn diagram of stroke topography for all patients (*n* = 101). **D**. Venn diagram of stroke topography for patients with repeated PSG (*n* = 72)

At admission, we evaluated demographics, anthropometrics, medical history, and stroke characteristics. All routine protocols were performed at identical field strengths and scanners of the same vendor (Siemens Healthineers, Germany) within 72 hours after stroke onset. Acute stroke topography was visually determined using diffusion-weighted imaging and FLAIR. It was coded by an expert reader blinded to clinical data for the involvement of any of four mutually exclusive parts of the brain: cerebral cortex (i.e., grey matter lesions), subcortical structures (i.e., lesions of subcortical hemispheric white matter, thalamus, and basal ganglia), brainstem and cerebellum. The cortical and subcortical categories formed the supratentorial category, whereas brainstem and cerebellum formed the infratentorial category. In case of multiple stroke locations, all affected areas were counted. Therefore, every patient had their stroke topography described with six binary-coded variables with one reflecting involvement of a given region in stroke, and null indicating no involvement (Figure1A).

A nocturnal PSG was performed in the acute phase (i.e., within 7 days after stroke) and at 3 months after stroke [17]. All PSGs were scored according to the AASM 2012 2.0 criteria. The following respiratory parameters were derived: AHI, obstructive apnea index (OAI), central apnea index (CAI), mixed apnea index (MAI), hypopnea index, oxygen desaturation index (ODI), time spent with oxygen saturation <90% (T90), and lowest oxygen saturation (LOS). Following the conservative approach, we did not distinguish the types of hypopneas (obstructive, central) [24]. Cheyne-Stokes-Respiration was described as a binary variable (yes/no) reflecting its presence according to visual scoring [18].

### Statistical analysis

Only patients with acute ischemic stroke who underwent both brain MRI and PSG at acute phase were included in the current analysis. Following the inspection of their distribution, some variables (e.g., AHI) were log-transformed. Missing values were not imputed. Continuous data were described as median and interquartile range, whereas categorical data were presented as number and percentages of total.

Continuous variables were compared between groups using the Wilcoxon rank-sum test. Independent categorical variables were compared using the Chi-square or Fisher’s exact test. We investigated the association between stroke topography and respiratory parameters at acute stroke and 3 months post-stroke using multiple linear regression with adjustment for confounders (e.g., age and sex). We investigated the change in respiratory sleep characteristics using the Wilcoxon signed-rank test (for continuous variables) and the McNemar test (for categorical variables). We analyzed the association of stroke topography with the change in respiratory parameters from acute stroke to 3 months post-stroke using multiple linear regression with adjustment for confounders.

All statistical tests were two-sided and conducted at the 5% significance level.

## Results

### Study population

Among 259 enrolled patients, 101 patients were included in the analysis (Supplementary figure 1). On average, the patients were 61.5 [54.8, 68.6] years old, were predominantly male (74.3%), and had minor stroke (NIHSS at admission: 2.0 [1.0, 5.0] points; Table 1). There were no differences in clinical characteristics between the patients with PSG at admission and patients with repeated PSG. There were no significant differences in stroke treatment (i.e., thrombolysis) between patients with SDB and those without, nor between patients with moderate-to-severe OSA and those without moderate-to-severe OSA. The prevalence and distribution of stroke lesions is shown in Figure 1B-1D.

**Table 1:** Demographic characteristics, stroke characteristics and clinical characteristics of SAS CARE 1 study subset (*n* = 101)

Parameter	All patients (*n* = 101)		Patients with repeated PSG at 3 months post-stroke (*n* = 72)		*P*-value
	*N*	Value	*N*	Value	
*Demographic characteristics*					
Age, years	101	61.5 [54.8, 68.6]	72	63.4 [56.4, 69.4]	0.406
Female sex	101	26 (25.7)	72	18 (25.0)	1
*Stroke characteristics*					
TOAST (%)	101		72		0.95^#^
Cardioembolism		23 (22.8)		18 (25.0)	
LAAS		10 (9.9)		6 (8.3)	
Other		22 (21.8)		14 (19.4)	
SVO		12 (11.9)		11 (15.3)	
Unknown		34 (33.7)		23 (31.9)	
Trombolysis at acute stroke	101	21 (20.8)	72	14 (19.4)	0.850
NIHSS at admission, points	101	2.00 [1.00, 5.00]	72	3.00 [1.00, 5.00]	0.854
mRS at discharge, points	98	1.00 [0.00, 2.00]	69	1.00 [0.00, 2.00]	0.77
mRS at 3 mps stroke, points	78	1.00 [0.00, 1.00]	56	1.00 [0.00, 1.00]	0.834
*Comorbidities*					
Hypertension	100	57 (57.0)	71	42 (59.2)	0.875
Coronary artery disease	101	16 (15.8)	72	11 (15.3)	1
Atrial fibrillation^1^	101	6 (5.9)	72	4 (5.6)	1
Heart failure	100	5 (5.0)	71	4 (5.6)	1
Diabetes	98	13 (13.3)	71	7 (9.9)	0.631
Dyslipidemia	100	72 (72.0)	71	48 (67.6)	0.612
Obesity	100	28 (28.9)	68	19 (27.9)	1
Smoking	101	29 (28.7)	72	19 (26.4)	0.863
Previous CCVE	101	4 (4.0)	72	1 (1.4)	0.403
Cheyne-Stokes-Respiration	101	10 (10.0)	72	7 (9.7)	1
*Sleep parameters*					
TST, hours	101	6.83 [5.56, 7.70]	72	6.42 [5.94, 7.38]	0.991
Arousal index,/h	101	14.90 [8.30, 23.30]	72	13.75 [8.88, 23.83]	0.983
Sleep efficiency, %	101	75.30 [61.40, 86.50]	72	76.40 [69.08, 86.70]	0.186
NREM1, %TST	101	11.95 [7.45, 21.83]	72	10.95 [5.88, 15.53]	0.036*
NREM2, %TST	101	51.70 [42.85, 58.75]	72	49.30 [42.60, 58.57]	0.748
NREM3, %TST	101	16.60 [9.30, 24.75]	72	19.25 [10.70, 27.20]	0.271
REM, %TST	101	15.95 [11.65, 20.80]	72	17.65 [14.50, 21.60]	0.064
AHI,/h	101	14.30 [7.00, 27.50]	72	6.50 [2.98, 21.80]	0.003*
AHI in NREM,/h	94	14.95 [6.60, 29.68]	52	12.40 [5.95, 25.20]	0.354
AHI in REM,/h	92	17.75 [7.12, 34.5]	51	19.70 [9.65, 34.35]	0.564
AHI supine,/h	93	24.60 [9.20, 58.00]	52	23.80 [8.22, 52.67]	0.924
Time in supine position, % TST	94	35.80 [13.32, 52.88]	52	48.55 [18.42, 64.40]	0.154
Time in supine position, hours	94	1.77 [0.79, 3.26]	52	2.67 [0.96, 3.74]	0.100
OAI,/h	101	2.20 [0.50, 7.40]	72	0.50 [0.08, 3.57]	0.001*
CAI,/h	101	0.20 [0.00, 1.20]	72	0.10 [0.00, 0.60]	0.130
MAI,/h	101	0.00 [0.00, 0.10]	72	0.00 [0.00, 0.20]	0.515
HI,/h	101	7.30 [3.50, 13.30]	72	4.55 [2.35, 14.57]	0.229
ODI,/h	93	8.25 [1.97, 20.62]	72	5.80 [2.28, 15.12]	0.327
T90, min	93	3.80 [0.12, 22.62]	72	3.70 [0.26, 15.82]	0.654
T90, %TST	93	1.01 [0.03, 6.05]	72	1.10 [0.01, 5.42]	0.990
LOS	93	86.00 [82.00, 89.00]	72	85.00 [81.00, 89.00]	0.883
*SDB severity*					
No SDB (AHI < 5/h)	101	16 (15.8)	72	11 (15.3)	0.724^#^
Mild SDB (5/h ≤ AHI < 15/h)		37 (37.0)		31 (43.1)	
Moderate SDB (15/h ≤ AHI < 30/h)		25 (12.9)		13 (18.1)	
Severe SDB (AHI ≥ 30/h)		23 (22.8)		17 (23.6)	
*SDB subtype (moderate and severe SDB)*^*1*^					
Obstructive SDB	101	37 (36.7)	72	24 (33.3)	1
Central SDB		11 (10.9)		6 (8.3)	

Continuous data is presented as median [interquartile range]. Categorical data is presented as count (% of total). Wilcoxon rank sum test was used for continuous variables. Fisher Exact Test and Chi-Square Test (^#^) were used for categorical variables. *p < 0.05

^1^At the time of stroke diagnosis. ^2^Moderate to severe SDB was divided into obstructive or central if OAI + MAI or CAI, respectively, was ≥ 50% of total apnea index

*AHI* apnea-hypopnea index, *CAI* central apnea index, *HI* hypopnea index, *LAAS* large artery atherosclerosis, *LOS* lowest oxygen desaturation, *MAI* mixed apnea index, *NREM* non-rapid eye movement, *OAI* obstructive apnea index, *SVO* small vessel occlusion, *CCVE* cardio-cerebrovascular event, *REM* rapid eye movement, *SDB* sleep-disordered breathing, *T90* time spent with oxygen saturation below 90%, *TST* total sleep time

72 patients (72%) underwent another PSG at 3 months post-stroke. The changes in sleep and respiratory parameters are shown in Supplementary Table 1 and Supplementary Figure 2.

### Respiratory sleep characteristics in different stroke topographies

We selected the patients with isolated supratentorial (n=78/98) or isolated infratentorial stroke (n=20/98) at the acute phase to minimize the confounding effect of lesions spreading across both areas (n=3).

**Fig. 2:**
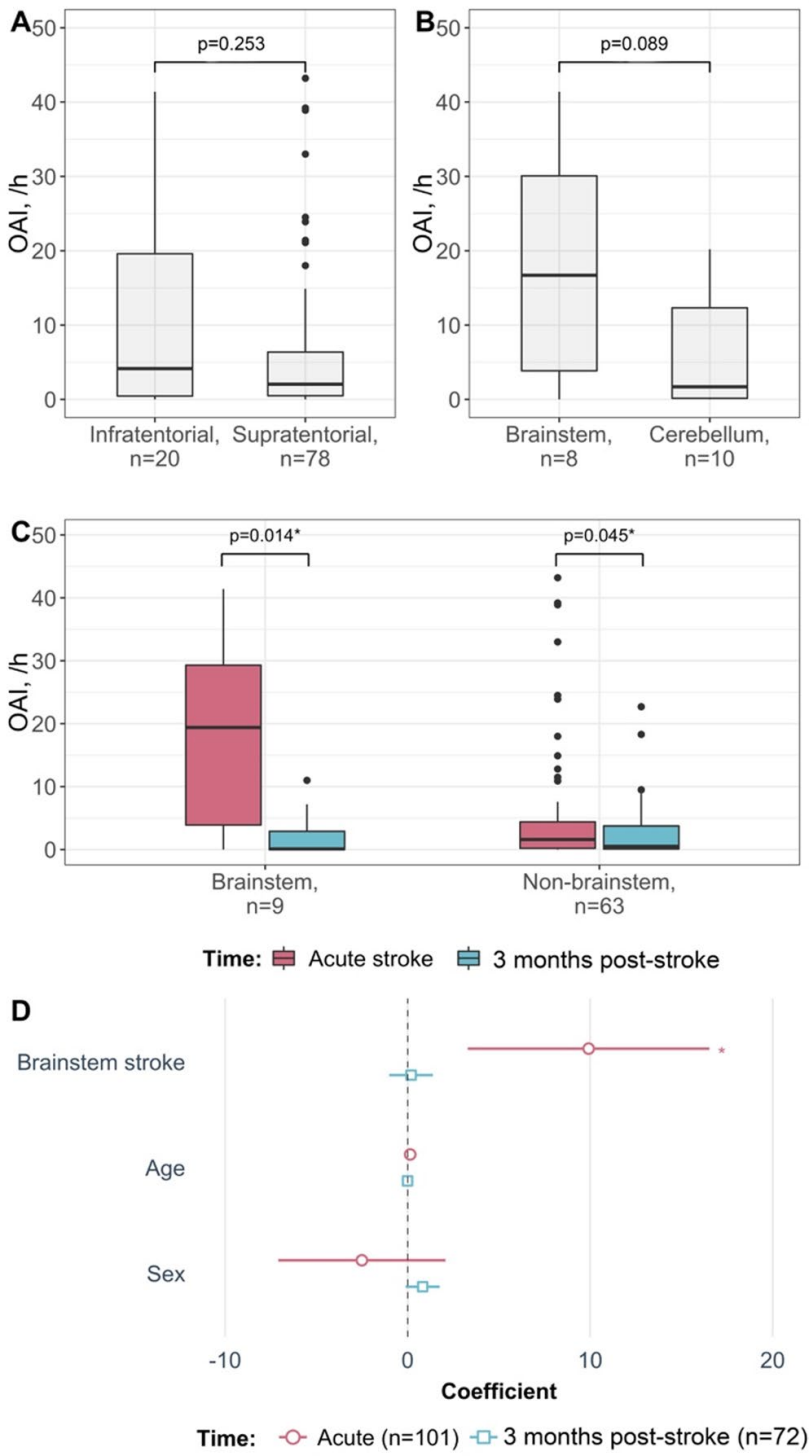
(**A**) OAI at acute stroke in isolated supratentorial (*n* = 78) compared to the isolated infratentorial (*n* = 20) stroke (Wilcoxon rank-sum test). (**B**) OAI at acute stroke in isolated brainstem (*n* = 8) compared to isolated cerebellar (*n* = 10) stroke (Wilcoxon rank-sum test). (**C**) Changes in OAI from acute stroke to 3 months post-stroke in patients with brainstem and non-brainstem lesion. (**D**) Coefficient plots of the multiple linear regression model showing the association of OAI with brainstem (*n* = 10/101 at acute stroke and *n* = 9/72 at 3 months post-stroke) versus non-brainstem lesion after adjustment for age and sex

There were no significant differences between OAI in isolated infratentorial stroke compared to supratentorial stroke (Figure2A). However, there was a positive association between any infratentorial stroke (n=23/101) and an OAI≥10/h (n=21/101, odds ratio=3.48, p=0.020).

We further compared OAI between the subgroups of patients with isolated infratentorial stroke. Among those, OAI in patients with isolated brainstem stroke (n=8/18) showed a trend to be higher compared to isolated cerebellar stroke (n=10/18; Figure 2B), but without reaching significance (p=0.089). This pattern was confirmed by the positive association between any brainstem stroke (n=10/101) with an OAI ≥10/h (n=21/101, odds ratio=4.59, p=0.030).

There were no significant differences in other respiratory parameters, including the presence of Cheyne-Stokes-Respiration or the presence and subtypes of moderate-to-severe SDB, between patients with the isolated supratentorial stroke versus the patients with isolated infratentorial stroke (Supplementary figure 3, Supplementary table 2).

### Association of stroke topography with respiratory sleep characteristics 

Given the pattern for differences in OAI in patients with brainstem stroke and the limited number of patients with isolated stroke, we binary-coded brainstem stroke topography to reflect the presence of non-isolated brainstem stroke (n=10/101) or the absence of brainstem stroke lesion (n=91/101). We then investigated the association between brainstem strokes and OAI at admission and at 3 months post-stroke after adjustment for demographics. The regression analysis confirmed the association of brainstem stroke with high OAI acutely after stroke (β=9.91 events/h, p=0.003). This association remained significant after additional adjustment for BMI, AHI in REM sleep, AHI in supine position and time in supine position (β=11.03 events/h, p<0.001). This association was not detectable at 3 months post-stroke (Figure 2D), most likely due to a marked OAI decrease from admission to 3 months post-stroke (1.95 [0.27, 6.95] and 0.50 [0.08, 3.57] /h, p<0.001, Supplementary table 1). Importantly, AHI in REM sleep, supine AHI, and time in supine position did not change significantly over time (Supplementary table 1).

The decrease of OAI from acute stroke to 3 months post-stroke was associated with brainstem (β=-10.72 events/h, p=0.011) stroke after adjustment for age and sex. After adjusting for baseline OAI, the mentioned association was no longer significant. The prevalence of brainstem lesions in moderate-to-severe SDB is reported in Supplementary table 2.

## Discussion

This observational study uses clinical MRI and PSG, the gold standards for diagnosing stroke and SDB, to investigate the association between stroke topography and SDB phenotype. This study is the largest PSG-based analysis in stroke patients to date. Additionally, we explore how stroke topography relates to the SDB evolution from the acute phase to 3 months post-stroke.

We showed an association between brainstem strokes and OAI during the acute stroke, based on early clinical MRI and PSG - the gold standards for diagnosing stroke and SDB. Our observation further supports physiological studies on the role of cranial nerves with brainstem nuclei in airway patency [19]. It also aligns with previous findings of higher OAI in acute infratentorial strokes (n=45) [11].

Our finding that brainstem stroke is linked to obstructive rather than central respiratory events contrasts a respiratory polygraphy study in acute stroke (n=355) reporting higher CAI and HI in brainstem infarction [5]. This discrepancy may stem from methodological differences: our study used MRI and PSG, while Brown et al. relied on radiology reports and respiratory polygraphy [5]. Additionally, brainstem centers *per se* (i.e., midbrain, pontine, and medullary) differentially affect respiratory drive and airway patency. The limited cases of brainstem stroke in both studies (11% vs. 10%) and differences in brainstem lesion topographies might also account for the observed discrepancies. Further research is needed to determine if specific brainstem lesion topography influences SDB phenotypes.

Furthermore, the lack of association between CAI or Cheyne-Stokes Respiration and stroke topography in this study contrasts with prior findings linking supratentorial and medullary lesions to central SDB [4, 20–23]. Cheyne-Stokes Respiration is characterized by periodic breathing patterns with cycles of apnea, whereas CSA involves a lack of respiratory effort. The mentioned discrepancy may result from low CAI values, a small number of central SDB cases, differing scoring methods, or varying prevalence of cardiac dysfunction across studies. Specifically, the predominance of mild strokes and the relatively limited number of patients with medullary strokes in our cohort may also have contributed to the low prevalence of central apneas, as more severe strokes are often associated with more complex SDB phenotypes [24].

This inconsistency may also be explained by the redundant respiratory control across the brain. Yuan et al. (2022) showed that lesions causing CSA share connectivity to the middle cingulate gyrus and bilateral cerebellar posterior lobes [25]. Future studies should explore subcortical lesion associations with CSA.

Since the association between brainstem lesions and high OAI was observed acutely but not at 3 months post-stroke, it is likely that the ischemic lesion itself primarily drives this relationship and that the recovery from the acute stroke may obscure the association over time. This pattern also suggests that obstructive apneas may represent an acute, transient manifestation of brainstem lesions rather than a persistent phenotype. Dysphagia, a common symptom after brainstem stroke, involves similar mechanisms controlling airway patency and swallowing. Notably, dysphagia often improves in the post-acute and chronic phases, and apneas may follow a comparable trajectory [26, 27]. Finally, the smaller sample size at 3 months (n=72 vs. n=101 at baseline) may further limit statistical power.

The strengths of our study include the prospective design with a comprehensive scoring of stroke topography and longitudinal PSG. While previous respiratory polygraphy-based studies had mostly a rather large sample size (n=355, n=74, n=116, n=106) [4–8], the sample size of previous polysomnography-based studies was limited (n=28, n=39, n=73, n=93) [11–14]. Among these studies, only three were longitudinal [6, 7, 11].

Our study has several limitations, including the lack of adjustment for stroke volume and cumulative lesion impact. Most patients in our cohort had a mild stroke, which limits the generalizability of our findings to the broad stroke population. While the sample size is larger than in other PSG-based acute stroke studies, distinct topographic groups are small and therefore the analysis can only provide hints about the directionality of effects. Overall, we consider the analysis underpowered, and it was done to explore the directionality of effects. Being observational, with unknown pre-stroke SDB levels, causality between stroke topography and OAI is assumed based on SDB improvement over 3 months, which contrasts its typical deterioration in the general population. Additionally, we do not differentiate hypopnea types, which limits the precision of our phenotyping. We did not characterize the relationship between electroencephalographic sleep features and stroke topography in sleep-disordered breathing, which will be addressed in future analyses.

In summary, our findings point to the potential involvement of brainstem lesions in the pathogenesis of obstructive apneas at acute stroke. Further studies should investigate the association of obstructive versus central SDB in brainstem stroke to further delineate specific regions potentially impacting the SDB phenotype. This knowledge may help identify stroke patients at risk for SDB who may benefit from potential treatment, improving post-stroke recovery. In this context, phenotyping patients with SDB, as well as the use of innovative home-based devices for diagnosis and treatment, may provide practical and scalable approaches to monitor and manage SDB, ultimately supporting improved post-stroke recovery [28–30].

## Supplementary Information

Below is the link to the electronic supplementary material.

ESM 1 (DOCX 527 KB)

## Data Availability

The data could be provided on a reasonable request to the corresponding author.

## Abbreviations

AASM: American Academy of Sleep Medicine
AHI: Apnea-hypopnea index
CAI: Central apnea index
CPAP: Continuous positive airway pressure
CSA: Central sleep apnea
DWI: Diffusion-weighted imaging
HI: Hypopnea index
LOS: Lowest oxygen saturation
MAI: Mixed apnea index
MRI: Magnetic resonance imaging
NIHSS: National Institutes of Health Stroke Scale
OAI: Obstructive apnea index
ODI: Oxygen desaturation index
OSA: Obstructive sleep apnea
PSG: polysomnography
RPG: respiratory polygraphy
SDB: Sleep-disordered breathing
T90: Time spent with oxygen saturation < 90%
