# Supplementary material for: SAS-CARE-1 study: stroke topography and polysomnography-based respiratory sleep characteristics
Source: Sleep Breath. 2025 Dec 5;30(1):4. doi: 10.1007/s11325-025-03540-8 (PMC12680655; doi:10.1007/s11325-025-03540-8)
Supplement: Supplementary file 1 — (DOCX 527 KB) [file 11325_2025_3540_MOESM1_ESM.docx]

## Supplementary figure 1. Study flowchart. Only patients with acute ischemic stroke who underwent both brain MRI and PSG at acute phase or at 3 months post-stroke in centers in Switzerland were included in the current analysis.


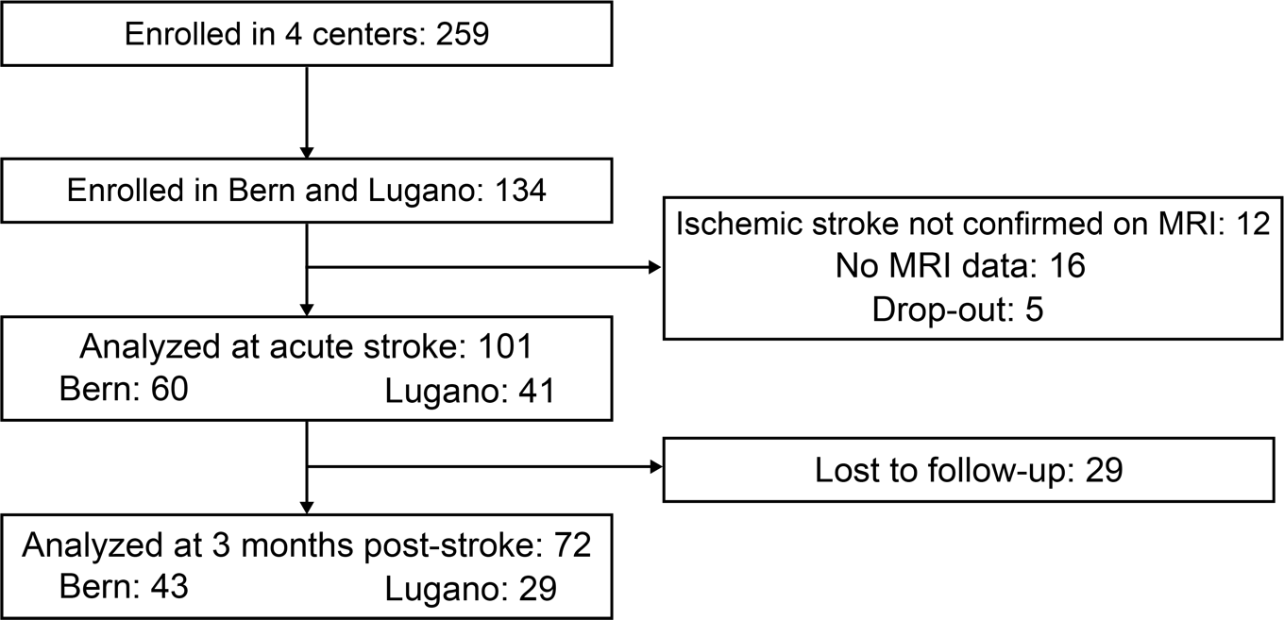


## Supplementary figure 2. Stacked chart^1^ of the changes in OAI and CAI from acute stroke to 3 months post-stroke. Overall, OAI and CAI remain stable in the majority of patients (68% and 88%, respectively). The OAI and CAI decrease in 25% and 8% of patients, respectively, whereas they increase in 10% and 4% of patients, respectively.

**
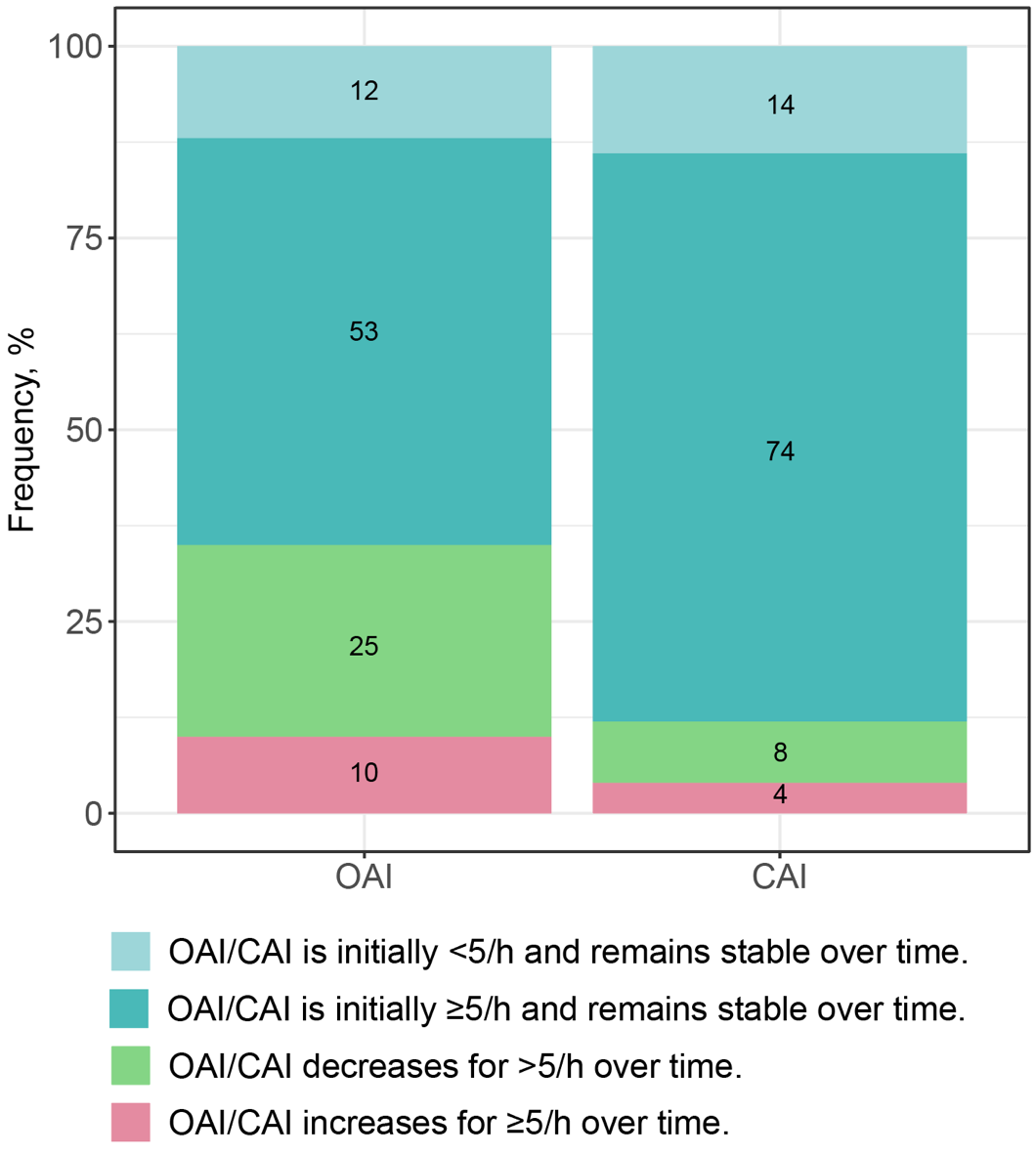
**

^1^Count is shown in the center of each bar section. Y-axis reflects the percentage of total.

CAI – central apnea index, OAI - obstructive apnea index.

## Supplementary figure 3. The differences in respiratory sleep characteristics between the patients with isolated supratentorial (n=78) compared to isolated infratentorial strokes (n=20). A. AHI. B. CAI. C. MAI. D. HI. E. T90. F. LOS.


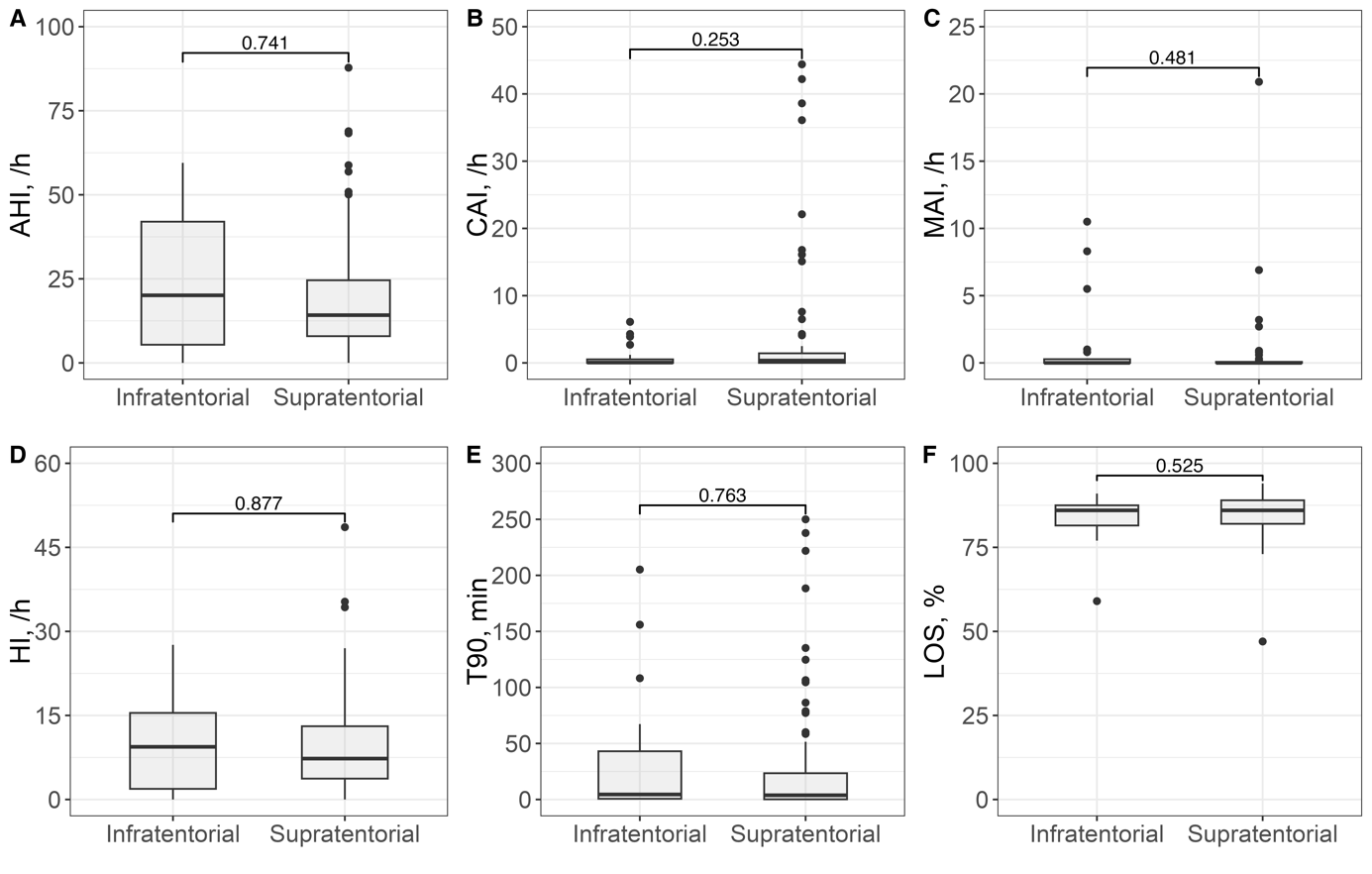


AHI – apnea-hypopnea index, CAI – central apnea index, HI – hypopnea index, MAI – mixed apnea index, OAI – obstructive apnea inex, ODI – oxygen desaturation index, T90 – time spent with oxygen saturation below 90%, LOS – lowest oxygen saturation.

## Supplementary table 1. Sleep and respiratory characteristics at acute stroke and at 3 months post-stroke.

| **Parameter** | **Acute stroke (n=101)** | **Acute stroke (n=72 with repeated assessment)** | **Repeated assessment at 3 months post-stroke** | **P-value** |
| --- | --- | --- | --- | --- |
| TST, hours | 6.83 [5.56, 7.70] | 6.60 [5.56, 7.66] | 6.42 [5.94, 7.38] | 0.574 |
| Arousal index, /h | 14.90 [8.30, 23.30] | 15.05 [8.30, 24.85] | 13.75 [8.88, 23.83] | 0.750 |
| Sleep efficiency, % | 75.30 [61.40, 86.50] | 73.25 [58.98, 85.92] | 76.40 [69.08, 86.70] | 0.078 |
| NREM1, %TST | 11.95 [7.45, 21.83] | 11.95 [7.25, 20.85] | 10.95 [5.88, 15.53] | 0.022* |
| NREM2, %TST | 51.70 [42.85, 58.75] | 52.20 [41.93, 59.55] | 49.30 [42.60, 58.57] | 0.915 |
| NREM3, %TST | 16.60 [9.30, 24.75] | 16.90 [11.70, 26.50] | 19.25 [10.70, 27.20] | 0.333 |
| REM, %TST | 15.95 [11.65, 20.80] | 14.75 [11.43, 18.97] | 17.65 [14.50, 21.60] | 0.106 |
| AHI, /h | 14.30 [7.00, 27.50] | 11.70 [6.68, 27.55] | 6.50 [2.98, 21.80] | 0.011* |
| AHI in NREM, /h | 14.95 [6.60, 29.68] | 14.95 [4.75, 30.92] | 12.40 [5.95, 25.20] | 0.316 |
| AHI in REM, /h | 17.75 [7.12, 34.50] | 17.20 [6.12, 34.55] | 19.70 [9.65, 34.35] | 0.986 |
| AHI supine, /h | 24.60 [9.20, 58.00] | 25.10 [9.20, 60.10] | 23.80 [8.22, 52.67] | 0.530 |
| Time in supine position, % TST | 35.80 [13.32, 52.88] | 32.80 [12.97, 52.85] | 48.55 [18.42, 64.40] | 0.149 |
| Time in supine position, hours | 1.77 [0.79, 3.26] | 1.59 [0.69, 3.03] | 2.67 [0.97, 3.75] | 0.147 |
| OAI, /h | 2.20 [0.50, 7.40] | 1.95 [0.27, 6.95] | 0.50 [0.08, 3.57] | 0.001* |
| CAI, /h | 0.20 [0.00, 1.20] | 0.10 [0.00, 1.00] | 0.10 [0.00, 0.60] | 0.048* |
| MAI, /h | 0.00 [0.00, 0.10] | 0.00 [0.00, 0.00] | 0.00 [0.00, 0.20] | 0.996 |
| HI, /h | 7.30 [3.50, 13.30] | 6.55 [3.30, 13.30] | 4.55 [2.35, 14.57] | 0.480 |
| ODI, /h | 8.25 [1.97, 20.62] | 7.80 [2.05, 20.60] | 5.80 [2.28, 15.12] | 0.070 |
| T90, min | 3.80 [0.12, 22.62] | 3.70 [0.05, 19.40] | 3.70 [0.26, 15.82] | 0.227 |
| T90, %TST | 1.01 [0.03, 6.05] | 1.10 [0.01, 5.42] | 1.10 [0.01, 5.42] | 0.515 |
| LOS | 86.00 [82.00, 89.00] | 86.00 [83.00, 88.75] | 85.00 [81.00, 89.00] | 0.806 |

Continuous data is presented as median [interquartile range]. Categorical data is presented as count (% of total). Wilcoxon signed-rank test was used for continuous variables. *p<0.05.

## Supplementary table 2. Prevalence of moderate-to-severe SDB in patients with supratentorial, infratentorial, and brainstem stroke.

| **Stroke** | **N** | **OSA** | **CSA** | **No SDB** | **P-value^2^** |
| --- | --- | --- | --- | --- | --- |
| Acute supratentorial stroke^1^ | 78/98 | 26 (33.3%) | 10 (12.8%) | 42 (53.8%) | 0.092 |
| Acute infratentorial stroke^1^ | 20/98 | 11 (55%) | 0 (0%) | 9 (45%) |  |
| Acute brainstem stroke | 10/101 | 6 (60%) | 0 (0%) | 4 (40%) | 0.187 |
| Subacute brainstem stroke | 9/72 | 2 (22.2%) | 1 (11.1%) | 6 (66.7%) | 0.664 |

Categorical data is presented as count (% of total).

^1^Of 98 patients with isolated supra- or infratentorial stroke. ^2^Chi-squared test: supratentorial versus infratentorial and brainstem stroke versus stroke, not affecting brainstem
